# Factors associated with requesting and receiving euthanasia: a nationwide mortality follow-back study with a focus on patients with psychiatric disorders, dementia, or an accumulation of health problems related to old age

**DOI:** 10.1186/s12916-019-1276-y

**Published:** 2019-02-19

**Authors:** Kirsten Evenblij, H. Roeline W. Pasman, Agnes van der Heide, Trynke Hoekstra, Bregje D. Onwuteaka-Philipsen

**Affiliations:** 1 Department of Public and Occupational Health, Amsterdam Public Health Research Institute, Amsterdam UMC, Vrije Universiteit Amsterdam, P.O. Box 7057, 1007 MB Amsterdam, The Netherlands; 2 Department of Public Health, Erasmus University Medical Center, Rotterdam, The Netherlands; 3 Department of Health Sciences, Amsterdam Public Health Research Institute, Vrije Universiteit Amsterdam, Amsterdam, The Netherlands

**Keywords:** Assisted suicide, Dementia, End-of-life care, Epidemiology, Euthanasia, Legislation, Medical decision-making, Policy, Psychiatry

## Abstract

**Background:**

Recently, euthanasia and assisted suicide (EAS) in patients with psychiatric disorders, dementia, or an accumulation of health problems has taken a prominent place in the public debate. However, limited is known about this practice. The purpose of this study was threefold: to estimate the frequency of requesting and receiving EAS among people with (also) a psychiatric disorder, dementia, or an accumulation of health problems; to explore reasons for physicians to grant or refuse a request; and to describe differences in characteristics, including the presence of psychiatric disorders, dementia, and accumulation of health problems, between patients who did and did not request EAS and between patients whose request was or was not granted.

**Methods:**

A nationwide cross-sectional survey study was performed. A stratified sample of death certificates of patients who died between 1 August and 1 December 2015 was drawn from the central death registry of Statistics Netherlands. Questionnaires were sent to the certifying physician (*n* = 9351, response 78%). Only deceased patients aged ≥ 17 years and who died a non-sudden death were included in the analyses (*n* = 5361).

**Results:**

The frequency of euthanasia requests among deceased people who died non-suddenly and with (also) a psychiatric disorder (11.4%), dementia (2.1%), or an accumulation of health problems (8.0%) varied. Factors positively associated with requesting euthanasia were age (< 80 years), ethnicity (Dutch/Western), cause of death (cancer), attending physician (general practitioner), and involvement of a pain specialist or psychiatrist. Cause of death (neurological disorders, another cause) and attending physician (general practitioner) were also positively associated with receiving euthanasia. Psychiatric disorders, dementia, and/or an accumulation of health problems were negatively associated with both requesting and receiving euthanasia.

**Conclusions:**

EAS in deceased patients with psychiatric disorders, dementia, and/or an accumulation of health problems is relatively rare. Partly, this can be explained by the belief that the due care criteria cannot be met. Another explanation is that patients with these conditions are less likely to request EAS.

## Background

Patients suffering unbearably may wish to hasten their death. Since 2002, the Netherlands has been one of the few countries where euthanasia and assisted suicide (EAS) is allowed under strict conditions [1]. The practice of EAS is restricted to physicians who must adhere to the “statutory due care criteria,” i.e., they must (1) be satisfied that the patient’s request is voluntary and well-considered; (2) be satisfied that the patient’s suffering is unbearable and without prospect of improvement; (3) have informed the patient about his situation and prognosis; (4) have come to the conclusion, together with the patient, that there is no reasonable alternative; (5) consult at least one other, independent physician; and (6) exercise EAS with due medical care and attention. Furthermore, the cause of suffering underlying the request must have a medical dimension, either somatic or psychiatric [1, 2], and physicians must report each case to the Regional Euthanasia Review Committees which review all EAS cases regarding whether the due care criteria were met.

In the past decade, the percentage of all deceased patients in the Netherlands who requested EAS prior to their death increased, from 5.2% in 2005, to 6.7% in 2011, and to 8.4% in 2015 [3]. Also, the percentage of requests that were carried out increased, from 37% in 2005, to 45% in 2010 and to 55% in 2015 [4]. Hence, not only is there a growing demand for EAS, requests are also more likely to result in EAS. Some evidence, however, suggests that requesting and receiving euthanasia depends, at least to some extent, on the cause of suffering. For instance patients who have cancer are more likely to request EAS compared to those with cardiovascular diseases [5]. Patients with physical symptoms, cancer, and a short life expectancy are more likely to receive EAS than others, while patients with depressive symptoms are less likely [6–8]. Also, demographic and care factors have been reported to influence requesting and receiving EAS [5–8].

Recently, EAS in patients with psychiatric disorders, dementia, or an accumulation of health problems related to old age (from now, accumulation of health problems) has taken a prominent place in the public debate [9–13]. In the Dutch Euthanasia Code, this last category, an accumulation of health problems, is referred to as a range of, mostly degenerative, disorders such as visual impairment, hearing impairment, osteoporosis, arthrosis, balance disorders, and cognitive decline [14]. Though the numbers are small, reports of the Euthanasia Review Committees have shown that the absolute number of EAS cases in people whose primary cause of suffering was a psychiatric disorder, dementia, or an accumulation of health problems has increased over the past 5 years [15–17].

Using a nationwide sample of deceased people, we studied requests for EAS in people with and without these conditions focusing on the following questions: How many deaths among people with psychiatric disorders, dementia, and accumulation of health problems were preceded by a request for EAS and how many of these requests were granted? What are the reasons to grant or refuse a request for EAS? Which patient and care characteristics, including the presence of psychiatric disorders, dementia, and an accumulation of health problems, are associated with a patient requesting EAS and with a patient receiving EAS?

## Methods

### Design and population

In 2015, a nationwide mortality follow-back study was performed to estimate the frequency of requesting and receiving EAS among people with (also) a psychiatric disorder, dementia, or an accumulation of health problems; to explore reasons for physicians to grant or refuse a request; and to describe differences in characteristics, including the presence of psychiatric disorders, dementia, and accumulation of health problems, between patients who did and did not request EAS and between patients whose request was or was not granted. The study was largely similar to previous mortality follow-back studies done in 1990, 1995, 2001, 2005, and 2010 [3, 4, 18–21]. A stratified sample of death certificates of persons who died between 1 of August and 1 of December 2015 was obtained from the central death registry of Statistics Netherlands. Death certificates were stratified into 10 strata based on the likelihood of the patient having made an end-of-life decision. The certifying physicians of the sampled cases received a questionnaire focusing on end-of-life decisions that might have preceded the death of the patient involved. A reminder was sent to those who had not returned the questionnaire. Of the 9351 questionnaires sent, 7277 were returned (response 78%). In this study, only those who died a non-sudden death and who were aged 17 years or older were included (*n* = 5361). Ethical approval was not required for the posthumous collection of anonymous patient data [22]. Further details of the study design are described elsewhere [3].

### Questionnaire

A four-page written questionnaire was sent to the physicians who signed the death certificates. The questionnaire was largely similar to the previous mortality follow-back studies [3, 4, 18–21]. It contained questions about the medical decision-making that had preceded death, whether the patient had requested for euthanasia, the reasons for granting or refusing the request, and questions about the medical care during the last month before death such as the involvement of caregivers for palliative consultation and psychosocial and spiritual issues. To obtain insight into EAS requests from people with a psychiatric disorder, dementia, and/or an accumulation of health problems (related to old age), a new question was added to the questionnaire about whether the patient had a psychiatric disorder, dementia, and/or an accumulation of health problems (yes/no). No description of these groups was provided to the physicians to classify patients; thus, physicians will most likely have interpreted these categories in the context of the Dutch euthanasia act and the current debate. The cause of death and specialty of the certifying physician were derived from the death certificate.

### Analysis

Statistical analyses were carried out using IBM SPSS version 22 (IBM Analytics). For presenting the frequencies of (requests for) EAS as well as the reasons for granting or refusing the requests, the results were made representative of all deaths during 2015 by weighting the data for stratification and response by patient’s sex, age, ethnic origin, and place and cause of death. This weighting procedure was similar to previous mortality follow-back studies [3, 4, 18–21]. Due to this procedure, the percentages that are reported cannot be derived from the absolute unweighted numbers.

Two multivariable logistic regression models were developed: one to identify factors associated with patients requesting EAS and one to identify factors associated with receiving EAS. The latter model was developed on a subset of the sample: patients who made an EAS request. First, the univariable association between each independent variable and the dependent variables (requesting EAS and receiving EAS) was analyzed. Next, all variables associated with requesting and receiving EAS (*p* value < 0.10) were entered in a multivariable model. Subsequently, a manual backward selection procedure was applied until only variables with *p* < 0.10 remained. In both models, the eligible independent variables were age (17–64, 65–79, > 80 years); sex (female/male); marital status (married/unmarried); ethnicity (Dutch and Western immigrants/non-Western immigrants); cause of death (cancer, cardiovascular disorder, pulmonary disorder, neurological disorder, or other); the presence of a psychiatric disorder (yes/no), dementia (yes/no), or an accumulation of health problems (yes/no); specialty of the certifying physician (general practitioner, medical specialist, or elderly care physician); involvement (yes/no) of the following caregivers in the last month of life, namely palliative care consultant/team, specialist pain control, psychiatrist/psychologist, and pastor [5–8]. Results are presented as frequencies, ORs, and 95% CIs.

### Extra analyses

In the multivariable model identifying factors associated with requesting EAS, the ORs and 95% CIs of cause of death changed drastically compared to the univariable model. Sensitivity analyses showed this was mainly driven by (i) collinearity between two variables, dementia and attending physicians; (ii) strong associations between cause of death, requesting EAS, and dementia and between cause of death, requesting EAS, and attending physician; and (iii) empty cells demonstrating the likelihood of unstable models. Therefore, we also performed the multivariable analyses for both requesting EAS and receiving EAS without dementia and attending physician. In these models, there was no indication for the issues described; the ORs and 95% CI of the variables did not change substantially compared to the univariable analyses. The results of the multivariable regression analyses including all independent variables (including dementia and attending physician) are reported as main outcomes.

## Results

### Description of the study sample

Of the 5361 deceased patients aged ≥ 17 years and whose death was non-sudden, 183 (3.4%) had a psychiatric disorder, 803 (15.0%) dementia, and 918 (17.1%) an accumulation of health problems, possibly next to the illness that caused their death. In people with a psychiatric disorder, dementia, or an accumulation of health problems, the most frequently reported cause of death was “other.” Of the people with dementia, 25.3% died of a neurological disorder (including dementia), and of the people with an accumulation of health problems, 22.1% died of a cardiovascular disorder. Among all deceased patients who died non-suddenly, 37% died due to cancer. The characteristics of the study sample are provided in Table 1.

**Table 1:** Characteristics of the sample stratified for psychiatric disorder, dementia, and/or an accumulation of health problems

	Psychiatric disorder Total *n* = 183		Dementia Total *n* = 803		Accumulation of health problems Total *n* = 918		All deceased patients who died non-suddenly Total *n* = 5361	
	*N*	%^1^	*N*	%^1^	*N*	%^1^	*N*	%^1^
Patient characteristics								
Sex								
Male	91	39.1	305	35.4	321	32.3	2672	45.9
Female	92	60.9	498	64.6	597	67.7	2689	54.1
Age								
17–64	63	19.7	7	0.8	2	0.0	1028	12.6
65–79	48	22.4	158	17.4	101	8.8	1936	30.6
80+	72	57.8	638	81.8	815	91.1	2397	56.8
Marital status								
Married	45	21.2	257	29.8	221	21.9	2538	41.7
Unmarried	138	78.8	546	70.2	697	78.1	2823	58.3
Ethnicity*								
Non-Western immigrants	27	3.7	74	1.5	87	1.9	605	2.9
Dutch, Western immigrants	133	96.3	729	98.5	829	98.1	4722	97.1
Cause of death								
Cancer	50	15.4	108	6.1	179	9.6	3128	37.1
Cardiovascular disorder	13	8.8	82	10.4	197	22.1	540	14.9
Pulmonary disorder	14	11.9	43	7.0	104	13.7	285	8.5
Neurological disorder	27	16.4	195	25.3	138	15.6	518	12.5
Other	79	47.5	375	51.2	300	38.9	890	27.0
Care characteristics								
Attending physician								
General practitioner	92	30.7	221	24.0	514	52.5	3301	50.0
Medical specialist	28	17.5	43	6.2	105	12.5	897	21.0
Elderly care physician	63	51.8	539	69.8	299	35.0	1163	29.1
Involvement of palliative care consultant/team								
No	164	91.5	761	95.4	842	92.5	4360	85.1
Yes	19	8.5	42	4.6	76	7.5	1001	14.9
Pain specialist								
No	178	98.5	801	99.7	905	98.9	5166	97.5
Yes	5	1.5	2	0.3	13	1.1	195	2.5
Psychiatrist/psychologist								
No	116	65.3	689	86.3	859	94.3	5050	93.8
Yes	67	34.7	114	13.7	59	5.7	311	6.2
Pastor								
No	156	82.3	676	84.4	782	84.6	4698	86.9
Yes	27	17.7	127	15.2	136	15.4	663	13.1

^1^Weighted column percentage. Deceased patients could have had a combination of psychiatric disorder, dementia, and/or an accumulation of health problems

*Missing *n* = 34 (0.6%)

### Frequency of EAS requests

**Fig. 1:**
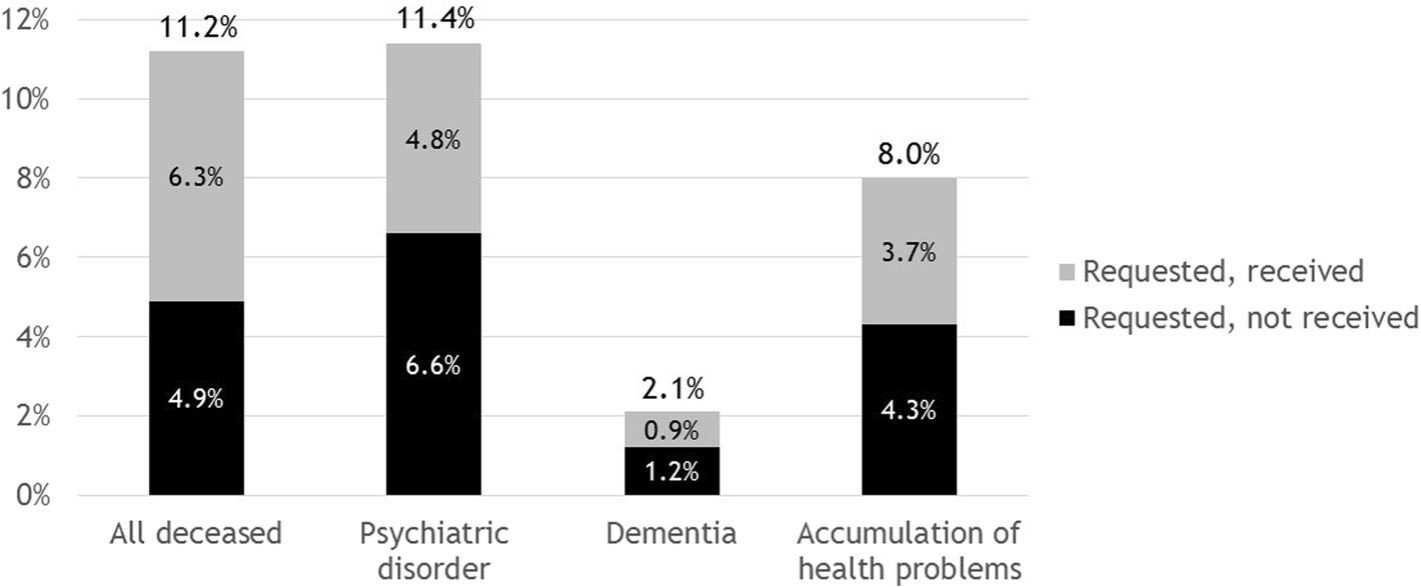
Frequency of deceased patients who did or did not receive euthanasia. Percentage of requests carried out among all deceased patients who died non-suddenly, 56% (6.3/11.2); people with psychiatric disorders, 42% (4.8/11.4); people with dementia 43% (0.9/2.1); and people with an accumulation of health problems, 46% (3.7/8.0)

Figure 1 shows that 11.2% of all patients who were aged ≥ 17 years had requested EAS preceding their death. Of the people with a psychiatric disorder, 11.4% requested EAS. The prevalence of EAS requests was lower among people with an accumulation of health problems (8.0%) and people with dementia requests (2.1%). Six percent of all deceased patients had received euthanasia; this percentage was lower among people who had a psychiatric disorder (4.8%), an accumulation of health problems (3.7%), and/or dementia (0.9%).

### Factors associated with requesting EAS

In univariable analyses, all variables showed associations (*p* < 0.10) with requesting EAS, except for the presence of a psychiatric disorder (Table 2). In the multivariable analysis, sex, marital status, and the involvement of a palliative care consultant were no longer associated (*p* < 0.10) with requesting EAS. Compared with people aged 80 years or older whose death was non-sudden, people aged between 17 and 64 years (OR 1.65 [1.33–2.04]) and between 65 and 79 (OR 1.38 [1.15–1.66]) were more likely to request EAS. Dutch and Western immigrants were 8.49 (95% CI 5.37–13.42) times more likely to request EAS compared with non-Western immigrants. Compared with people who died of cancer, people who died of cardiovascular disorders were less likely to request EAS while people who died of pulmonary disorders, neurological disorders, or another cause were more likely. People with an accumulation of health problems (OR 0.69 [0.53–0.90]) or dementia (OR (0.18 [0.12–0.28]) had lower odds of requesting EAS compared with those without these conditions. People whose attending physician was a medical specialist or an elderly care specialist had lower odds of requesting EAS (OR 0.07 [0.05–0.11] and OR 0.17 [0.13–0.23]) compared with people whose attending physician was a general practitioner. People who were supported by pain specialists (OR 2.08 [1.47–2.93]) and psychiatrists (OR 4.50 [3.15–6.41]) in the last month of life were more likely to request EAS while those supported by pastors were less likely (OR 0.77 [0.59–1.00]).

**Table 2:** Factors associated to requesting EAS (people above the age of 16 whose death was non-sudden)

	Absolute number in sample	No EAS request	EAS request	Univariable logistic regression		Multivariable logistic regression		Sensitivity analysis	
	*N* = 5361	*N* = 4243 Row %^†^	*N* = 1118 Row %^†^	Odds ratio (95% CI)	*p*	Odds ratio (95% CI)	*p*	Odds ratio (95% CI)	*p*
Patient characteristics									
Sex									
Male	2672	87.1	12.9	Reference					
Female	2689	90.3	9.7	0.84 (0.74–0.96)	0.011	–		–	
Age									
17–64	1028	80.2	19.8	2.29 (1.92–2.73)	< 0.001	1.65 (1.33–2.04)	< 0.001	2.13 (1.75–2.59)	< 0.001
65–79	1936	86.0	14.0	1.82 (1.56–2.12)	< 0.001	1.38 (1.15–1.66)	0.001	1.59 (1.35–1.88)	< 0.001
80+	2397	92.2	7.8	Reference		Reference		Reference	
Marital status									
Married	2538	85.9	14.1	Reference					
Unmarried	2823	90.9	9.1	0.71 (0.63–0.81)	< 0.001	–		–	
Ethnicity^‡^									
Non-Western immigrants	605	96.9	3.1	Reference		Reference			
Dutch, Western immigrants	4722	88.6	11.4	8.36 (5.38–12.98)	< 0.001	8.49 (5.37–13.42)	< 0.001	9.24 (5.91–14.44)	< 0.001
Cause of death									
Cancer	3128	81.1	18.9	Reference		Reference		Reference	
Cardiovascular disorders	540	94.2	5.8	0.29 (0.22–0.40)	< 0.001	0.64 (0.46–0.89)	0.009	0.40 (0.30–0.55)	< 0.001
Pulmonary disorders	285	87.7	12.3	0.77 (0.57–1.03)	0.077	1.94 (1.37–2.76)	< 0.001	0.95 (0.70–1.29)	0.772
Neurological disorders	518	94.3	5.7	0.57 (0.45–0.73)	< 0.001	1.85 (1.37–2.51)	< 0.001	0.69 (0.53–0.89)	0.004
Other	890	94.2	5.8	0.42 (0.34–0.52)	< 0.001	1.42 (1.08–1.88)	0.012	0.55 (0.43–0.69)	< 0.001
A psychiatric disorder									
No	5178	88.8	11.2	Reference		NE		NE	
Yes	183	88.6	11.4	0.99 (0.69–1.43)	0.976				
An accumulation of health problems									
No	4443	87.7	12.3	Reference		Reference			
Yes	918	92.0	8.0	0.50 (0.40–0.61)	< 0.001	0.69 (0.53–0.90)	0.005	–	
Dementia									
No	4558	86.0	14.0	Reference		Reference			
Yes	803	97.9	2.1	0.13 (0.09–0.19)	< 0.001	0.18 (0.12–0.28)	< 0.001	NE	
Care characteristics									
Attending physician^§^									
General practitioner	3301	81.2	18.8	Reference		Reference			
Medical specialist	897	96.7	3.3	0.09 (0.07–0.13)	< 0.001	0.07 (0.05–0.11)	< 0.001	NE	
Elderly care physician	1163	96.1	3.9	0.14 (0.10–0.18)	< 0.001	0.17 (0.13–0.23)	< 0.001	NE	
Care givers involved in the last month of life									
Palliative care consultant/team									
Not involved	4360	90.1	9.9	Reference					
Involved	1001	81.6	18.4	1.43 (1.22–1.68)	< 0.001	–	–	–	
Specialist pain control									
Not involved	5166	89.3	10.7	Reference		Reference		Reference	
Involved	195	69.7	30.2	2.31 (1.71–3.11)	< 0.001	2.08 (1.47–2.93)	< 0.001	1.82 (1.32–2.50)	< 0.001
Psychiatrist/psychologist									
Not involved	5050	89.1	10.9	Reference		Reference		Reference	
Involved	311	84.6	15.4	1.44 (1.11–1.86)	0.006	4.50 (3.15–6.41)	< 0.001	2.05 (1.54–2.73)	< 0.001
Pastor									
Not involved	4698	88.5	11.5	Reference		Reference		Reference	
Involved	663	90.9	9.1	0.55 (0.44–0.70)	< 0.001	0.77 (0.59–1.00)	0.050	0.53 (0.42–0.68)	< 0.001

– indicates the item was entered in the regression but *p* > 0.10 and consequently eliminated in the stepwise procedure; NE indicates the item was not entered in the regression

†Weighted row percentage

‡34 missing (0.6%)

The results of the extra analysis without the variables dementia and attending physician (see the “Methods” section) were largely similar to the original multivariable model. However, people who died of cancer were now more likely to request EAS compared to people who died of any other cause. An accumulation of health problems dropped from the model.

### Reasons to grant or refuse the request

Table 3 shows that across the full sample, the two most important reasons for the attending physician to grant the request were the lack of prospect of improvement (81.9–94.6%) and the autonomy of the patient (72.4–85.8%). In case of a psychiatric disorder, the presence of (severe) symptoms other than pain (75.4%) and expected suffering (53.5%) were also important reasons. In case of dementia, the loss of dignity (73.7%) and expected suffering of the patient (49.1%) were important. Finally, in case of an accumulation of health problems, the presence of symptoms other than pain (48.7%) and loss of dignity (54.8%) were both important reasons to grant the request. Among those with a psychiatric disorder, dementia, or an accumulation of health problems, the most important reason to refuse the request was that the due care criteria were not met, especially regarding the well-considered nature of the request. Among all deceased patients, the most important reason was that the patient died before the request was granted.

**Table 3:** Reasons for either or not granting the EAS request stratified for psychiatric disorder, dementia, and/or an accumulation of health problems

	Deceased with a psychiatric disorder (*n* = 183) %^1^	Deceased with dementia (*n* = 803) %^1^	Deceased with an accumulation of health problems (*n* = 918) %^1^	All deceased patients who died non-suddenly (*n* = 5361) %^1^
Reasons for the physician to grant the request and perform euthanasia*	*N* = 24	*N* = 22	*N* = 80	*N* = 845
No prospect of improvement	87.3	94.6	83.4	81.9
Autonomy of the patient	85.8	72.4	81.0	80.7
(Severe) symptoms other than pain	75.4	26.2	48.7	61.2
Loss of dignity	32.0	73.7	54.8	59.1
(Severe) pain	20.3	12.6	34.9	40.4
Expected suffering of the patient	53.5	49.1	30.9	44.3
Further treatment would be too burdensome	21.6	21.4	22.2	14.5
Other	11.0	15.5	4.1	1.8
Reasons for the request not resulting in euthanasia*	*N* = 14	*N* = 9	*N* = 36	*N* = 273
Patient died before the request could be granted	13.0	8.1	23.5	53.1
The criteria for due care were not met*	44.4	76.1	70.6	32.1
No well-considered request	34.8	59.8	32.4	16.2
No unbearable suffering	18.1	16.3	40.5	12.0
No hopeless suffering	16.8	16.3	13.5	5.1
No voluntary request	0	4.3	0	0.7
Generally	8.3	10.1	5.6	4.3
Patient withdrew the request	18.7	13.9	15.7	17.4
Physician never willing to perform euthanasia	0	0	5.5	2.1
Other	29.0	21.5	14.7	9.4

^1^Weighted column percentage

*More than one answer possible

### Factors associated with receiving EAS

Table 4 shows associations between receiving EAS and patient and care characteristics. In univariable analyses, age, cause of death, the presence of a psychiatric disorder and an accumulation of health problems, attending physician, and the involvement of a palliative care consultant/team and pastor showed associations (*p* < 0.10) with receiving EAS. In multivariable analysis, most associations remained significant. People who died of neurological disorders or another cause had 4.70 [95% CI 2.09–10.58] and 2.38 [95% CI 1.34–4.26] times higher odds of receiving EAS compared with people who died of cancer. People with a psychiatric disorder and an accumulation of health problems had lower odds of receiving EAS compared with people without these conditions (OR 0.38 [0.18–0.82] and OR 0.62 [0.36–1.05]). People whose attending physician was a medical specialist or an elderly care specialist were less likely to receive EAS (OR 0.13 [0.06–0.27] and OR 0.16 [0.09–0.28]) compared with people whose attending physician was a general practitioner. Those who were supported by a palliative care consultant in the last month of life were also less likely to receive EAS (OR 0.70 [0.50–0.98]).

**Table 4:** Factors associated with receiving EAS (people above the age of 16 whose death was non-sudden)

	Absolute number in the sample	Request did not result in EAS	Request did result in EAS	Univariable logistic regression		Multivariable logistic regression		Sensitivity analysis	
	*N* = 1118	*N* = 273%^1^	*N* = 845%^1^	Odds ratio (95% CI)	*p*	Odds ratio (95% CI)	*p*	Odds ratio (95% CI)	*p*
Patient characteristics									
Sex									
Male	595	42.9	57.1	Reference					
Female	523	44.7	55.3	1.14 (0.87–1.50)	0.349	NE		NE	
Age									
17–64	294	42.1	57.9	1.07 (0.76–1.53)	0.690	–		–	
65–79	467	38.3	61.7	1.32 (0.96–1.81)	0.093	–		–	
80+	357	50.0	50.0	Reference					
Marital status									
Married	604	44.7	55.3	Reference					
Unmarried	514	42.8	57.2	1.21 (0.92–1.59)	0.184	NE		NE	
Ethnicity									
Non-Western immigrants	21	25.0	75.0	Reference					
Dutch, Western immigrants	1091	43.8	56.2	1.27 (0.49–3.31)	0.622	NE		NE	
Cause of death									
Cancer	808	40.7	59.3	Reference		Reference		Reference	
Cardiovascular disorders	50	65.3	34.7	0.56 (0.31–1.01)	0.054	0.72 (0.37–1.41)	0.332	0.70 (0.37–1.35)	0.291
Pulmonary disorders	60	57.6	42.4	0.87 (0.48–1.55)	0.628	1.53 (0.77–3.03)	0.221	1.02 (0.55–1.90)	0.942
Neurological disorders	86	22.5	77.5	3.34 (1.58–7.03)	0.002	4.70 (2.09–10.58)	< 0.001	3.92 (1.81–8.47)	0.001
Other	114	45.5	54.5	1.35 (0.83–2.20)	0.220	2.38 (1.34–4.26)	0.003	1.83 (1.06–3.14)	0.029
A psychiatric disorder									
No	1080	43.3	56.7	Reference		Reference		Reference	
Yes	38	56.5	43.5	0.54 (0.28–1.06)	0.074	0.38 (0.18–0.82)	0.013	0.38 (0.18–0.79)	0.010
An accumulation of health problems									
No	1002	41.7	58.3	Reference		Reference		Reference	
Yes	116	54.1	45.9	0.69 (0.45–1.05)	0.081	0.62 (0.36–1.05)	0.073	0.63 (0.38–1.04)	0.070
Dementia									
No	1087	43.1	56.9	Reference					
Yes	31	59.3	40.7	0.78 (0.36–1.72)	0.545	NE		NE	
Care characteristics									
Attending physician									
General practitioner	1016	37.8	62.2	Reference		Reference			
Medical specialist	36	66.7	33.3	0.13 (0.06–0.27)	< 0.001	0.13 (0.06–0.27)	< 0.001	NE	
Elderly care physician	66	79.4	20.6	0.19 (0.12–0.32)	< 0.001	0.16 (0.09–0.28)	< 0.001	NE	
Care givers involved in the last month of life									
Palliative care consultant/team									
Not involved	872	42.3	57.7	Reference		Reference		Reference	
Involved	246	48.4	51.6	0.65 (0.48–0.89)	0.007	0.70 (0.50–0.98)	0.037	0.65 (0.48–0.90)	0.008
Specialist pain control									
Not involved	1046	43.4	56.6	Reference					
Involved	72	48.8	51.2	0.90 (0.52–1.54)	0.688	NE		NE	
Psychiatrist/psychologist									
Not involved	1034	42.8	57.2	Reference					
Involved	84	54.7	45.3	0.75 (0.46–1.21)	0.237	NE		NE	
Pastor									
Not involved	1029	42.1	57.9	Reference					
Involved	89	58.2	41.8	0.49 (0.31–0.77)	0.002	–		0.49 (0.30–0.78)	0.003

– indicates the item was entered in the regression but was not significant (> 0.10) and consequently eliminated in the stepwise procedure; NE indicates the item was not entered in the regression

†Weighted row percentage

‡6 missing (0.5%)

The results of the extra analysis without the variables dementia and attending physician (see the “Methods” section) were largely similar to the original multivariable model except for the negative association found between pastor and receiving EAS.

## Discussion

The frequency of EAS requests among deceased people who died non-suddenly and who had psychiatric disorders (11.4%), dementia (2.1%), and/or an accumulation of health problems (8.0%) varied. Less than half of these requests led to EAS. Factors positively associated with requesting EAS were age (< 80 years), ethnicity (Dutch/Western), cause of death (cancer), attending physician (general practitioner), and involvement of pain specialist and psychiatrist. Cause of death (neurological disorders or another cause) and attending physician (general practitioner) were also positively associated with receiving euthanasia. Psychiatric disorders, dementia, and accumulation of health problems were negatively associated with requesting and receiving EAS.

### EAS in people with psychiatric disorders, dementia, and an accumulation of health problems

EAS in people with psychiatric disorders, dementia, and an accumulation of health problems is a highly debated subject, but this practice rarely occurs. Partially, this can be explained by reluctance of physicians to perform EAS in these patients [23]. Our results showed that the proportion of euthanasia requests that was carried out was lower among people with psychiatric conditions (42%), dementia (43%), and an accumulation of health problems (46%) compared to all non-sudden deceased people (56%). Moreover, having a psychiatric disorder or an accumulation of health problems was statistically significantly associated with a lower likelihood of having a request being carried out. Previous research has also shown that physicians consider it less likely to perform EAS in patients with a psychiatric disorder, dementia, and/or an accumulation of health problems compared to patients with a severe and life-limiting somatic illness such as cancer [23–25]. Our results suggest that the presence of a psychiatric disorder, dementia, and/or an accumulation of health problems may complicate the decision to grant a request, even if the patient also suffers from a severe and life-limiting somatic illness, such as cancer. The main reasons to refuse a request are doubts about whether the request was well-considered and about the unbearableness of the suffering. These findings corroborate previous studies [26, 27].

This study is the first to show that people with dementia or an accumulation of health problems are less likely to request EAS compared to people without these conditions which may explain part of the lower frequency of EAS in people with these conditions. Possibly, the lower frequency of requests among people with dementia and an accumulation of health problems can be explained by the slow and gradual decline characterizing both dementia and an accumulation of health problems leading to the gradual acceptance of a declining health condition [28–30]. In addition, in case of advanced dementia, patients lose the ability to make a well-considered request for EAS.

Due to the aging society, associated with an increasing number of older people suffering from multimorbidity, it is likely that the number of EAS requests from patients suffering from dementia and/or an accumulation of health problems related to old age will continue to grow [31, 32]. The question of how policy makers and care providers should respond to these requests is, therefore, highly relevant.

### Characteristics associated with requesting and receiving EAS

#### Patient characteristics

This study showed that younger people are more likely to request EAS which is consistent with previous studies in the Netherlands and Belgium [5, 6, 33]. Younger people tend to have more permissive and liberal attitudes compared to older people and are more likely to support EAS [34, 35]. Also, a strong positive association between ethnicity and requesting EAS was found, with Dutch or Western migrants being 8.5 times more likely to request EAS compared to non-Western migrants. Cultural and religious values and beliefs have frequently been reported to profoundly influence the perceptions of death and end-of-life decision-making [36–40].

People who died due to a neurological disorder were almost four times more likely to receive EAS compared to people with cancer which corresponds with previous findings [5, 33, 41]. ALS disease, which is known for its progressive, severe physical symptoms and lack of effective treatments, probably contributes the most to this finding.

#### Care characteristics

The involvement of a pain specialist and the involvement of a psychiatrist/psychologist in the last month of life were associated with higher likelihood of requesting EAS. This confirms previous research in Belgium and the Netherlands [5, 42]. Possibly, pain specialists and psychiatrists/psychologists stimulate patients to think and talk about their end-of-life wishes, including EAS, as autonomy and informed decision-making are key principles of palliative care [43]. Finally, prior to granting a request, a physician must be certain that there is no other reasonable solution; optimizing end-of-life care is one of them.

Multivariable regression analyses also showed that deceased patients who were attended by a general practitioner were more likely to request and receive EAS, supporting previous evidence [5]. The attendance of a general practitioner possibly provides more opportunity for discussing end-of-life wishes, including euthanasia, due to the long-term care relationship with the patient and the non-acute care setting.

### Strengths and limitations

Major strengths of this study are the large nationwide sample which is the representative of all deaths in the Netherlands in 2015, the high response rate and few missing data. When interpreting the results, some limitations need to be considered. Physicians were asked whether the patient had either one or more of the following conditions: a psychiatric disorder, dementia, and an accumulation of health problems. Since this was a general, closed question, i.e., yes/no, it is unknown to what extent these conditions contributed to the suffering underlying the EAS request. Also, psychiatric disorders and an accumulation of health problems are very broad categories which one has to take into account when interpreting the results. Another limitation is that our sample included patients who were seriously ill after all our sample included deceased patients; patients without a life-threatening illness were not included unless their life was ended. On the one hand, this may have led to an underestimation of the number of requests since among those who request EAS are also people who are not seriously ill. On the other hand, it may have led to an overestimation of the number of requests granted among people with a psychiatric disorder, dementia, and/or an accumulation of health problems since physicians are more likely to grant requests of people with (also) a severe and life-limiting somatic condition.

## Conclusions

A relatively small group of people who died non-suddenly received EAS but even fewer of those with (also) psychiatric disorders, dementia, or an accumulation of health problems. Partly, this can be explained by the belief that the due care criteria cannot be met. Another explanation is that patients with these conditions are less likely to request for it. Given the aging society and the related rising of the number of EAS requests from people suffering from dementia and/or an accumulation of health problems, the question of how policy makers and care providers should respond to these requests is highly relevant.

## Abbreviations

Accumulation of health problems: Accumulation of health problems related to old age
CI: Confidence interval
EAS: Euthanasia and assisted suicide
OR: Odds ratio
